# Eligibility of Outpatients with Chronic Heart Failure for Vericiguat and Omecamtiv Mecarbil: From Clinical Trials to the Real-World Practice

**DOI:** 10.3390/jcm14061951

**Published:** 2025-03-13

**Authors:** Paolo Basile, Alessio Falagario, Maria Cristina Carella, Marco Maria Dicorato, Francesco Monitillo, Daniela Santoro, Maria Ludovica Naccarati, Gianluca Pontone, Marco Matteo Ciccone, Vincenzo Ezio Santobuono, Andrea Igoren Guaricci

**Affiliations:** 1 Cardiology Unit, Interdisciplinary Department of Medicine, University of Bari “Aldo Moro”, Polyclinic University Hospital, 70121 Bari, Italy; paolo.basile@uniba.it (P.B.); a.falagario2@studenti.uniba.it (A.F.); m.carella31@phd.uniba.it (M.C.C.); m.dicorato20@studenti.uniba.it (M.M.D.); dr.francescomonitillo@gmail.com (F.M.); danina2012@gmail.com (D.S.); marialudovica97@libero.it (M.L.N.); marcomatteo.ciccone@uniba.it (M.M.C.); vincenzoezio.santobuono@uniba.it (V.E.S.); 2 Department of Perioperative Cardiology and Cardiovascular Imaging, IRCCS Centro Cardiologico Monzino, 20138 Milan, Italy; gianluca.pontone@cardiologicomonzino.it; 3 Department of Biomedical, Surgical and Dental Sciences, University of Milan, 20122 Milan, Italy

**Keywords:** chronic heart failure, vericiguat, omecamtiv mecarbil, guidelines-directed medical therapy

## Abstract

**Background**: Several drugs are emerging as potential therapeutic resources in the context of chronic heart failure (CHF), although their impact on daily clinical practice remains unknown. The objective of this study was to investigate the theoretical eligibility for vericiguat and omecamtiv mecarbil (OM) in a real-world outpatient setting. **Methods**: A cross-sectional observational study was conducted, enrolling all patients with CHF who had at least one visit between January 2023 and January 2024 in a dedicated outpatient clinic of a tertiary referral center. Theoretical eligibility for vericiguat and OM in our population was assessed by adopting the criteria of the respective phase III clinical trials (VICTORIA trial for vericiguat and GALACTIC-HF trial for OM). **Results**: In 350 patients with CHF, the rate of individuals eligible was 2% for vericiguat and 4% for OM. A value for left ventricular ejection fraction (LVEF) over the clinical trials’ cutoffs was observed in 41% of cases for vericiguat and 69% for OM. The absence of a recent heart failure (HF) worsening was found in 78% of cases for vericiguat and 72% for OM. **Conclusions**: Only a small proportion of CHF patients would be eligible for vericiguat and OM in a real-world outpatient setting. The absence of a recent HF worsening and an LVEF over the established trials’ cutoffs are the main causes of non-eligibility. Further studies are required to assess the efficacy of these drugs in a wider population in order to increase the candidates for these beneficial treatments.

## 1. Introduction

Chronic heart failure (CHF) remains a major cause of mortality and a significant public health concern on a global scale [1,2,3,4,5,6,7,8]. In recent years, advancements in imaging and laboratory techniques have facilitated enhanced diagnostic support of CHF [9,10,11,12]. Moreover, technological innovation has developed new devices for a more precise monitoring of clinical conditions [13,14,15,16]. In the near future, artificial intelligence tools will provide further improvements in the management of these patients [17,18,19,20,21,22]. However, the most significant advancements were obtained in the therapeutic field, with the advent of novel drugs that have demonstrated to improve the prognosis and the quality of life of these patients [23,24,25,26,27]. Recently, the SGLT2 inhibitors have revolutionized the therapeutic scenario of CHF thanks to their beneficial effects on the cardiovascular system and high safety [28,29,30,31,32,33,34]. In particular, these antidiabetic drugs were demonstrated to improve prognosis also in patients with CHF and preserved left ventricular ejection fraction (LVEF), a specific and large group of patients without treatments capable of significantly changing their prognosis until not so long ago [35,36,37]. Other innovative medications, which have yet to be widely adopted in routine therapeutic management, are emerging as promising resources [38,39,40,41]. Among these, the most relevant are vericiguat and omecamtiv mecarbil (OM). The former is an oral soluble Guanylate Cyclase (sGC) stimulator, a class of drugs widely adopted in the treatment of pulmonary hypertension and, for the first time, considered in worsening CHF [42,43,44,45,46,47]. The VICTORIA trial, a large phase III trial, demonstrated that vericiguat may reduce cardiovascular death and HF hospitalizations compared to placebo in CHF patients with reduced LVEF and a recent hospitalization (hazard ratio (HR) = 0.90; 95% confidence interval (CI) = 0.82 to 0.98; *p* = 0.02) [48]. OM, a cardiac myosin activator, represents a drug acting on an unexplored target with the potential to improve myocardial function augmenting cardiac sarcomere function in patients with severe CHF [49,50,51,52]. A beneficial effect of OM in CHF population with reduced LVEF was observed in a large phase III trial, the GALACTIC-HF, demonstrating a lower incidence of a composite outcome, including an HF event or cardiovascular death, compared to placebo (HR = 0.92; 95% CI = 0.86 to 0.99; *p* = 0.03) [53]. The full extent of the potential impact of these innovative drugs on clinical practice needs to be further explored. The objective of this study was to evaluate the rate of theoretical eligibility for vericiguat and OM in patients with CHF in a real-world outpatient setting, according to eligibility criteria adopted in the respective phase III clinical trials [48,53].

## 2. Materials and Methods

A cross-sectional observational study was conducted, enrolling all patients with CHF who had at least one visit between January 2023 and January 2024 in a dedicated clinic of a tertiary referral center. The study was performed in accordance with the principles of the Declaration of Helsinki and was approved by the Local Ethics Committee (Protocol Number 58102 by Comitato Etico Territoriale Azienda Ospedaliero Universitaria Consorziale Policlinico di Bari). Written informed consent was obtained from each patient before enrolment in the study. For each patient, we collected anamnestic, clinical, echocardiographic, and laboratory data. The diagnosis of CHF was made according to the definitions outlined in the updated European guidelines [23,24]. The eligibility of the participants was assessed by evaluating, for each patient, the presence of inclusion and exclusion criteria of the respective phase III trials (VICTORIA trial for vericiguat and GALACTIC HF for OM) [48,53]. More specifically, we adopted the following criteria of the respective phase III clinical trials:

- VICTORIA trial—inclusion criteria: Patients aged 18 years or older with symptomatic (New York Heart Association (NYHA) class II-IV) CHF on standard therapy, with LVEF <45% and a previous HF hospitalization within 6 months or IV diuretic treatment for HF (without hospitalization) within 3 months, increased B-type natriuretic peptide (BNP) and amino terminal pro-brain natriuretic peptide (NT-proBNP) values. Exclusion criteria: Clinically unstable patients; systolic blood pressure <100 mmHg or symptomatic hypotension; use of long-acting nitrates/nitric oxide (NO) donors, phosphodiesterase type 5 (PDE5) inhibitors, or sGC stimulators; patients awaiting heart transplantation or receiving continuous IV infusion of inotrope or implanted ventricular assist device; presence of primary valvular heart disease requiring or within 3 months after surgery/intervention; hypertrophic obstructive cardiomyopathy; acute myocarditis; amyloidosis; sarcoidosis; takotsubo cardiomyopathy post-heart transplant cardiomyopathy; tachycardia-induced cardiomyopathy and/or uncontrolled tachyarrhythmia; acute coronary syndrome or coronary revascularization within 60 days or indication for coronary revascularization; symptomatic carotid stenosis; transient ischemic attack (TIA) or stroke within 60 days; complex congenital heart disease; active endocarditis or constrictive pericarditis; estimated glomerular filtration rate (eGFR) <15 mL/min/1.73 m^2^ or chronic dialysis; severe hepatic insufficiency; malignancy or other non-cardiac condition limiting life expectancy to <3 years; continuous home oxygen for severe pulmonary disease; current alcohol and/or drug abuse; interstitial lung disease; and pregnancy or breastfeeding [48].
- GALACTIC HF trial—inclusion criteria: Patients ≥ 18 to ≤85 years of age, symptomatic (NYHA class II to IV) CHF and LVEF ≤ 35%, managed with HF SoC therapies with a recent (within 1 year) hospitalization for heart failure (HHF) or urgent visit to emergency department (ED) for heart failure, and increased BNP or NT-proBNP levels. Exclusion criteria: Patients with malignancies (latest 5 years); mechanical hemodynamic support; IV treatment with inotropes, vasopressors, diuretics, or vasodilators; supplemental oxygen therapy; non-invasive mechanical ventilation; recent (within 3 months) acute coronary syndrome, stroke, or TIA; recent cardiac surgery or percutaneous interventions; recent (30 days) insertion of other cardiac devices; severe uncorrected valvular heart disease; hypertrophic or infiltrative cardiomyopathy, active myocarditis; constrictive pericarditis; clinically significant congenital heart disease; untreated severe arrhythmias; systolic blood pressure > 140 mmHg or <85 mmHg, or diastolic blood pressure > 90 mmHg, or heart rate > 110 beats per minute, or < 50 beats per minute; eGFR < 20 mL/min/1.73 m^2^ or receiving dialysis; hepatic impairment; and pregnancy or breastfeeding [53].

This process was conducted independently by two cardiologists with extensive experience in the management of CHF (F.M. and D.S.). Any discrepancies were resolved through discussion and consensus or adjudication from a third senior cardiologist (A.I.G.). All statistical analysis were conducted using SPSS version 25 software (SPSS, Inc., Chicago, IL, USA). For continuous variables, the normal distribution was evaluated using the Shapiro–Wilk test and, in the case of a normal distribution, expressed as mean and standard deviation. Otherwise, they were described in terms of median and interquartile range (IQR). They were compared using the two-tailed t-test for independent variables in the case of normal distribution; otherwise, they were compared using the Mann–Whitney U-test. The data of the categorical variables were presented as frequencies (percentage) and compared with χ2 test or Fisher’s exact test, when appropriate. For eligibility rates of vericiguat and OM, we calculated the relative 95% confidence intervals for a single proportion.

## 3. Results

A total of 350 patients were enrolled in the study, with a median age of 67 years (interquartile range (IQR), 17 years) and a clear predominance of male sex (77% of cases). The prevalence of cardiovascular risk factors is notable, particularly arterial hypertension and dyslipidemia, which were observed in 66% and 63% of cases, respectively. Approximately one-third of the population was obese. Chronic kidney disease (CKD) was a prevalent comorbidity, affecting 35% of cases. Of these, only a small proportion (1.4%) was undergoing dialysis. With regard to the clinical evaluation, a majority of patients were in NYHA classes II and III (80%). A high percentage of patients were treated with guidelines-directed medical therapy. In particular, beta-blockers were taken by 93% of our population, and ACE inhibitors/angiotensin II receptor blockers or angiotensin receptor neprilysin inhibitors were taken by 84%. Lower rates were observed for mineralocorticoid receptor antagonists (67%) and SGLT2 inhibitors (55%). A total of 70% of patients received loop diuretics for symptom control. The main characteristics of the study population are displayed in Table 1.

### 3.1. Eligibility for Vericiguat

**Figure 1:**
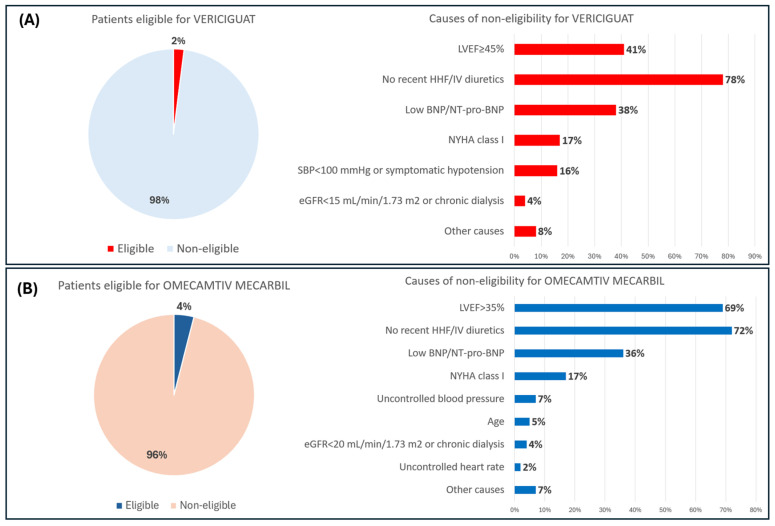
Theoretical eligibility for vericiguat and omecamtiv mecarbil. (**A**) Eligibility for vericiguat and the principal reasons for ineligibility, as determined by the VICTORIA trial. (**B**) Eligibility for omecamtiv mecarbil and the main causes of non-eligibility according to the GALACTIC HF trial. LVEF = left ventricular ejection fraction; HHV = hospitalization for heart failure; IV = intravenous; BNP = B-type natriuretic peptide; NT-proBNP = amino terminal pro-brain natriuretic peptide; NYHA = New York Heart Association; SBP = systolic blood pressure; eGFR= estimated glomerular filtration rate.

Upon applying the criteria of the VICTORIA trial to our population, it was found that the percentage of individuals eligible for vericiguat was 2.0% (IC 95% = 0.8 to 4.0%). The most significant reason for non-eligibility was the absence of a recent (i.e., within 6 months) HHF or use of IV diuretics (i.e., within 3 months) found in 78% of patients. In 12% of cases, we observed an HHF or treatment with IV diuretics, despite occurring beyond the time limits set by the trial. Another frequent cause of exclusion (41% of cases) was the presence of an LVEF of ≥45%. Other contributing factors included the presence of atrial natriuretic peptide (BNP/NT-pro-BNP) values below the established trial cutoff and a NYHA class I (38% and 17%, respectively), as shown in Figure 1.

### 3.2. Eligibility for Omecamtiv Mecarbil

The eligibility of patients for OM was evaluated in accordance with the inclusion and exclusion criteria of the GALACTIC HF trial. The rate of patients eligible for this drug was 3.7% (IC 95% = 2.0 to 6.3). The most important cause of non-eligibility was the absence of recent HHF/IV diuretics, identified in 72% of cases. Another significant factor, found in 69% of patients, was the presence of an LVEF over the established trial cutoff (35%). Other causes included the presence of atrial natriuretic peptide values below the predicted cutoffs and NYHA class I (36% and 17% of cases, respectively), as shown in Figure 1.

### 3.3. Comparison of Eligible and Non-Eligible Patients for Vericiguat and OM

Although the analysis may have been affected by the large discrepancy in the sample size of the two groups, interesting differences emerge between the two groups. All eligible patients have a recent HHF with higher values of natriuretic peptides compared to non-eligible patients (*p* = 0.012 for vericiguat and *p* = 0.001 for OM). An interesting finding, although not statistically significant, is the presence of a higher number of eligible patients with signs of congestion, requiring more frequently the use of diuretic therapy and higher daily doses of loop diuretics. Moreover, the percentage of eligible patients with a recent dosage increase in diuretic therapy tends to be higher, although non-statistically significant (*p* = 0.418 for vericiguat and *p* = 0.100 for OM). Regarding echocardiographic data, eligible patients show lower values of LVEF (*p* = 0.002 for vericiguat and *p* < 0.001 for OM), a more dilated left ventricle (LVESV, *p* = 0.035 for vericiguat and *p* < 0.001 for OM) and a tendency toward a more severe diastolic dysfunction with higher rates of E/A > 2 and more prominent mitral and tricuspid regurgitations. The results of this analysis are shown in Table 1.

## 4. Discussion

Vericiguat and OM are two novel drugs with the potential to positively impact the clinical outcome of CHF [48,53,54,55,56]. Their mechanism of action differs from that of currently available treatments. Vericiguat represents the first oral sGC activator approved for symptomatic chronic HFrEF in several countries, including Europe [57]. This drug, stimulating sGC enzymes, part of the NO-sGC-cyclic guanosine monophosphate signaling pathway, proved beneficial effects on both the myocardial and vascular endothelial cells [58,59,60,61]. In the VICTORIA trial, it was pointed out that vericiguat compared to placebo induced a reduction in mortality due to cardiovascular causes or HHF in patients with LVEF < 45%, elevated natriuretic peptide concentrations, and a recent HF worsening [48]. In light of these encouraging outcomes, the latest European guidelines now recommend the use of this drug for the management of CHF in adults with reduced LVEF who have stabilized following a recent HHF or IV therapy diuretics [23,24]. A large registry of 23,573 HF patients in Sweden estimated that 21.4% of patients would be eligible for vericiguat according to the criteria of the VICTORIA trial, while a higher proportion (47.4%) could be eligible according to European guidelines [62]. In a further large-scale Korean study of 3014 patients with HFrEF and a recent rehospitalization, the eligibility rate was 95% in accordance with the FDA label criteria, while a lower percentage (58%) met the criteria of the VICTORIA study [63]. Similar results were also pointed out by Moghaddam et al. [64]. These findings, obtained in patients discharged from hospital after HHF, were at odds with the proportions of eligible patients observed in our study, which was conducted in an outpatient setting. Indeed, the most frequent exclusion criterion in our population was the absence of a recent HHF episode, which is a well-known predictive factor for mortality in patients with CHF [65,66,67,68]. We have postulated that the discrepancy in eligibility rates may be attributed to the different settings in which these studies were conducted. Moreover, in our population, a considerable proportion of patients were treated using the four disease-modifying drugs recommended by European guidelines, thereby reducing the risk of further episodes of HHF. In fact, in previous studies, including the VICTORIA trial, SGLT2 inhibitors were not considered, although they demonstrated the capability to reduce the risk of worsening CHF [28,31,69]. Therefore, our data suggest that the presence of a recent HHF episode could be a limiting factor in the large-scale use of this drug in an outpatient setting. In the near future, the results of the ongoing VICTOR trial (NCT05093933), testing the effects of vericiguat in CHF patients without recent HHF, may represent a turning point in this context [70]. OM is a selective cardiac myosin activator that has not yet been approved for clinical use. Its mechanism of action provides a new therapeutic target that is completely different from currently used drugs, based upon the improvement of myocardial contractility by the increase in the number of cardiac myosin heads linked to actin filaments [71,72,73,74]. The phase III trial GALACTIC HF pointed out a lower incidence of heart-failure events in HFrEF patients treated with OM compared to placebo [53]. Another important observation emerging from our data is that a significant exclusion criterion both for vericiguat and OM was an LVEF over the established trials cutoffs. Indeed, data from large registries have indicated that only approximately 50% of patients were classified with HFrEF [75,76,77]. Preliminary studies pointed out conflicting results on the role of these drugs in patients with preserved LVEF requiring further large-scale investigations with longer follow-up [78,79,80]. Moreover, we should emphasize the potential role that vericiguat and OM could play in certain high-risk subcategories of CHF patients, such as advanced NYHA class or subjects with CKD, as suggested by recent evidence [81].

### Study Strengths and Limitations

To the best of our knowledge, for the first time, we provide real-world data on potential eligible patients for vericiguat and OM in an outpatient setting. This analysis could provide useful insights to guide further studies, with the aim of increasing the number of patients who could benefit from these drugs. The monocentric design and the low sample size represent important limitations that should be taken into account in the interpretation and generalization of these results. A selection bias related to missing data may have influenced the eligibility rates and should be considered in the evaluation of these results.

## 5. Conclusions

The findings of this study indicate that a limited proportion of patients diagnosed with CHF would meet the criteria for prescription of these novel drugs in a real-world outpatient context. The absence of recent HHF/IV diuretics and LVEF over the established trials cutoffs are the primary causes of non-eligibility. Further studies are required to assess the efficacy of these drugs in the population of HF patients excluded by clinical trials in order to increase the candidates for these beneficial treatments.

## Figures and Tables

**Table 1:** Baseline characteristics of the study population and a comparison between eligible and non-eligible patients for vericiguat and OM. CVD = cardiovascular disease; ACS = acute coronary syndrome; PCI = percutaneous coronary intervention; CABG = coronary artery bypass grafting; TIA = transient ischemic attack; OHT = orthotopic heart transplantation GDMT = guidelines-directed medical therapy; ACEI = angiotensin-converting enzyme inhibitor; ARB = angiotensin II receptor blocker; ARNI = angiotensin receptor-neprilysin inhibitor; MRA = mineralocorticoid receptor antagonist; SGLT2i = sodium-glucose co-transporter-2 inhibitors; PDE5 = phosphodiesterase-5; HHF = heart failure hospitalization; CIED = cardiac implantable electronic device; BMI = Body Mass Index; NYHA = New York Heart Association; HR= heart rate; SBP = systolic blood pressure; DBP = diastolic blood pressure; eGFR = estimated glomerular filtration rate; AST = aspartate transaminase; ALT = alanine aminotransferase; NT-proBNP= N-terminal pro-b-type natriuretic peptide; IVS = interventricular septum; LVEDD = left ventricular end-diastolic diameter; LVEDV = left ventricular end-diastolic volume; LVESV = left ventricular end-systolic volume; LAAPD = left atrium antero-posterior diameter; SPAP = systolic pulmonary artery pressure; TAPSE = tricuspid annular plane systolic excursion; IVC = inferior vena cava; MR = mitral regurgitation; TR = tricuspid regurgitation. Bold has been used to differentiate statistically significant *p*-values.

Baseline Parameters	Total (n = 350)	Eligible for Vericiguat (n = 7)	Non-Eligible for Vericiguat (n = 343)	*p*-Value	Eligible for Omecamtiv (n = 13)	Non-Eligible for Omecamtiv (n = 337)	*p*-Value
Age, median [IQR]	67 [17]	72 [16]	67 [17]	0.485	72 [17]	67 [17]	0.383
Sex male, n (%)	270 (77.1)	6 (85.7)	264 (77.0)	0.585	11 (84.6)	259 (76.9)	0.513
Family history of CVD, n (%)	94 (26.9)	1 (14.3)	93 (27.2)	0.446	3 (23.1)	91 (27.1)	0.749
Arterial hypertension, n (%)	232 (66.5)	4 (57.1)	228 (66.7)	0.691	6 (46.2)	226 (67.3)	0.114
Dyslipidemia, n (%)	220 (63.2)	3 (42.9)	217 (63.6)	0.267	6 (46.2)	214 (63.9)	0.193
Diabetes Mellitus, n (%)	111 (32.1)	3 (42.9)	108 (31.9)	0.685	4 (30.8)	107 (32.1)	0.918
Smoking habit, n (%)	163 (46.7)	3 (42.9)	160 (46.8)	1.000	6 (46.2)	157 (46.7)	0.968
Obesity, n (%)	116 (33.3)	2 (28.6)	114 (33.4)	1.000	3 (23.1)	113 (33.7)	0.424
Ischemic etiology, n (%)	137 (39.1)	4 (57.1)	133 (38.8)	0.439	9 (69.2)	128 (38.0)	**0.024**
Alcohol/drugs abuse, n (%)	9 (2.6)	0 (0.0)	9 (2.6)	0.664	0 (0.0)	9 (2.6)	0.890
Chronic Kidney Disease, n (%)	123 (35.2)	3 (42.9)	120 (35.1)	0.701	7 (53.8)	116 (34.5)	0.152
Chronic dialysis, n (%)	5 (1.4)	0 (0.0)	5 (1.5)	1.000	0 (0.0)	5 (1.5)	1.000
Hepatic insufficiency, n (%)	3 (0.9)	0 (0.0)	3 (0.9)	1.000	0 (0.0)	3 (0.9)	1.000
Interstitial Lung Disease, (%)	22 (6.3)	0 (0.0)	22 (6.3)	0.488	0 (0.0)	22 (6.5)	0.341
ACS/PCI/CABG/STROKE/TIA, n (%)	149 (42.7)	3 (42.9)	146 (42.7)	1.000	8 (61.5)	141 (42.0)	0.162
Active cancer, n (%)	16 (4.6)	0 (0.0)	16 (4.7)	0.558	0 (0.0)	16 (4.8)	0.421
Enrolment in transplant list, n (%)	17 (4.9)	0 (0.0)	17 (4.9)	0.546	0 (0.0)	17 (4.9)	0.483
OHT, n (%)	9 (2.6)	0 (0.0)	9 (2.6)	0.664	0 (0.0)	9 (2.6)	0.551
GDMT							
– ACEI/ARB/ARNI, n (%)	294 (84.2)	7 (100)	287 (83.9)	0.248	13 (100)	281 (95.6)	0.112
– Beta blockers, n (%)	324 (92.8)	7 (100)	317 (97.8)	0.458	13 (100)	311 (96.0)	0.307
– MRA, n (%)	235 (67.5)	6 (85.7)	229 (67.2)	0.435	12 (92.3)	223 (66.6)	0.052
– SGLT2i, n (%)	193 (55.8)	6 (85.7)	187 (55.2)	0.139	11 (84.6)	182 (54.7)	**0.033**
– Diuretics, n (%)	242 (69.5)	7 (100)	235 (68.9)	0.106	12 (92.3)	230 (68.7)	0.069
Diuretics daily dose (mg/die), median [IQR]	25 [50]	50 [75]	25 [25]	0.502	62.5 [75]	25 [25]	0.128
Long-acting nitrates, n (%)	1 (0.3)	0 (0.0)	1 (0.3)	1.000	0 (0.0)	1 (0.3)	1.000
PDE5 inhibitors, n (%)	0 (0.0)	0 (0.0)	0 (0.0)	-	0 (0.0)	0 (0.0)	-
IV infusion of inotrope, n (%)	3 (0.9)	0 (0.0)	3 (0.9)	1.000	0 (0.0)	3 (0.9)	1.000
Oxygen therapy, n (%)	2 (0.6)	0 (0.0)	2 (0.6)	1.000	0 (0.0)	2 (0.6)	1.000
Lipid lowering therapy, n (%)	265 (75.9)	5 (71.4)	260 (76.0)	0.778	11 (84.6)	254 (75.6)	0.455
Anticoagulants, n (%)	188 (53.9)	4 (57.1)	184 (53.8)	1.000	9 (4.8)	179 (53.3)	0.257
Anti-arrhythmic drugs, n (%)	125 (35.8)	1 (14.3)	124 (36.3)	0.429	5 (38.5)	120 (35.7)	0.839
CIED, n (%)	264 (75.9)	6 (85.7)	258 (75.7)	0.538	13 (100)	251 (74.9)	**0.038**
Recent HHF, n (%)	45 (12.9) vericiguat—64 (18.3) OM	7 (100)	38 (11.1)	**<0.001**	13 (100)	51 (15.1)	**<0.001**
**Clinical parameters**							
BMI (Kg/m2), median [IQR]	27.0 [6.9]	26.0 [8.0]	27.0 [6.9]	0.327	24.0 [5.0]	28.0 [6.0]	**0.010**
NYHA class, n (%)				0.635			0.229
I	60 (17.2)	0 (0.0)	60 (17.5)		0 (0.0)	60 (17.9)	
II	166 (47.6)	4 (57.1)	162 (47.4)		6 (46.2)	160 (47.6)	
III	116 (33.2)	3 (42.9)	113 (33.0)		7 (53.8)	109 (32.4)	
IV	7 (2.0)	0 (0.0)	7 (2.0)		0 (0.0)	7 (2.1)	
Pulmonary congestion, n (%)	36 (10.3)	0 (0.0)	36 (10.3)	0.631	1 (7.7)	35 (10.4)	0.876
Jugular turgor, n (%)	14 (4.0)	0 (0.0)	14 (4.1)	0.583	0 (0.0)	14 (4.2)	0.450
Peripheral oedema, n (%)	70 (20.2)	2 (28.6)	68 (20.1)	0.579	7 (53.8)	63 (18.9)	**0.002**
Hepatomegaly, n (%)	14 (4.0)	0 (0.0)	14 (4.1)	0.583	1 (7.7)	13 (3.9)	0.490
Cold extremities, n (%)	1 (0.3)	0 (0.0)	1 (0.3)	1.000	0 (0.0)	1 (0.3)	1.000
Recent diuretic dose increase, n (%)	23 (6.7)	1 (14.3)	22 (6.5)	0.418	6 (46.2)	86 (25.7)	0.100
Signs of vascular congestion, n (%)	92 (26.4)	2 (28.6)	90 (25.9)	0.897	2 (15.4)	21 (6.4)	0.202
HR (bpm), median (IQR)	67 [15]	60 [6]	67 [15]	0.153	60 [9]	67 [15]	0.103
SBP (mmHg), median [IQR]	110 [20]	110 [45]	110 [20]	0.525	100 [23]	110 [20]	0.438
DBP (mmHg), median [IQR]	70 [15]	80 [20]	70 [10]	0.322	60 [20]	70 [15]	0.697
Atrial fibrillation, n (%)	166 (47.6)	1 (14.3)	165 (48.2)	0.124	6 (46.2)	160 (47.6)	0.917
**Laboratory parameters**							
Creatinine (mg/dl), median [IQR]	1.1 [0.5]	1.0 [0.5]	1.3 [0.5]	0.573	1.3 [0.9]	1.1 [0.5]	0.527
eGFR (ml/min/m2), median [IQR]	64.7 [38.0]	70.0 [30.0]	64.5 [38.0]	0.638	53.0 [37.7]	65.0 [38.0]	0.524
Bilirubin (mg/dl), median [IQR]	0.70 [0.44]	1.20 [0.00]	0.69 [0.45]	0.174	0.92 [0.96]	0.70 [0.44]	0.529
AST (UI), median [IQR]	22 [10]	25 [15]	22 [10]	0.603	29 [12]	21 [10]	0.172
ALT (UI), median [IQR]	20 [15]	30 [0]	20 [15]	0.319	31 [0]	20 [15]	0.260
BNP (pg/mL), median [IQR]	79 [105]	-	79 [105]	-	-	79 [105]	-
NTproBNP (pg/mL), median [IQR]	1115 [2354]	2202 [6095]	1023 [2310]	**0.012**	2721 [5884]	930 [2236]	**0.001**
**Echocardiographic parameters**							
IVS (mm), median (IQR)	11 [3]	10 [4]	11 [3]	0.221	10 [3]	11 [3]	**0.002**
LVEDD (mm), median (IQR)	55 [12]	55 [18]	55 [12]	0.193	63 [15]	55 [11]	**0.002**
LVEDV (mL), median (IQR)	127 [62]	173 [150]	127 [62]	0.175	217 [156]	125 [61]	**0.001**
LVESV (mL), median (IQR)	73 [55]	133 [120]	73 [54]	**0.035**	177 [139]	73 [51]	**<0.001**
LVEF (%), median (IQR)	41 [19]	25 [8]	41 [18]	**0.002**	23 [18]	41 [18]	**<0.001**
LAAPD (mm), median (IQR)	44 [10]	46 [7]	44 [10]	0.844	47 [9]	44 [10]	0.128
E/A > 2, n (%)	29 (11.4)	2 (33.3)	27 (10.8)	0.086	2 (20.0)	27 (11.0)	0.381
E/E’, median [IQR]	9.5 [4.9]	8.5 [11.8]	9.5 [4.9]	0.825	10.5 [11.8]	9.5 [4.9]	0.379
SPAP (mmHg), median [IQR]	29 [10]	35 [27]	29 [10]	0.456	35 [14]	29 [10]	**0.039**
TAPSE (mm), median [IQR]	19 [5]	18 [8]	19 [5]	0.526	19 [6]	19 [5]	0.557
S’ tricuspidal, median [IQR]	10.7 [3.0]	9.7 [4.0]	10.7 [3.0]	0.531	11.5 [3.2]	10.6 [3.0]	0.783
IVC diameter (mm), median [IQR]	16 [4]	17 [4]	16 [4]	0.442	16 [6]	16 [4]	0.618
IVC collapse, n (%)	294 (87.5)	6 (85.7)	288 (87.5)	0.885	11 (84.6)	283 (87.6)	0.748
MR moderate or more, n (%)	81 (23.6)	2 (28.6)	79 (23.6)	0.318	6 (46.2)	75 (22.7)	0.080
TR moderate or more, n (%)	72 (21.0)	3 (42.9)	69 (20.6)	0.517	6 (46.2)	66 (19.9)	0.082

## Data Availability

The data that support the findings of this study are available from the corresponding author upon reasonable request.

## Abbreviations

CHF	chronic heart failure
LVEF	left ventricular ejection fraction
OM	omecamtiv mecarbil
sGC	soluble guanylate cyclase
NYHA	New York Heart Association
NO	nitric oxide
PDE5	phosphodiesterase type 5
HHF	hospitalization for heart failure
ED	emergency department
TIA	transient ischemic attack
eGFR	estimated glomerular filtration rate
IQR	interquartile range
CKD	chronic kidney disease
BNP	B-type natriuretic peptide
NT-pro-BNP	amino terminal pro-brain natriuretic peptide
